# Does the Use of Learning Management Systems With Hypermedia Mean Improved Student Learning Outcomes?

**DOI:** 10.3389/fpsyg.2019.00088

**Published:** 2019-02-12

**Authors:** María Consuelo Sáiz-Manzanares, Raúl Marticorena-Sánchez, José Francisco Díez-Pastor, César Ignacio García-Osorio

**Affiliations:** ^1^Faculty of Health Sciences, Hospital Militar, University of Burgos, Burgos, Spain; ^2^Department of Health Science, University of Burgos, Burgos, Spain; ^3^Department of Civil Engineering, University of Burgos, Burgos, Spain

**Keywords:** hypermedia resources, Moodle, Smart Tutoring System, learning outcomes, Educational Data Mining

## Abstract

Learning management systems (LMSs) that incorporate hypermedia Smart Tutoring Systems and personalized student feedback can increase self-regulated learning (SRL), motivation, and effective learning. These systems are studied with the following aims: (1) to verify whether the use of LMS with hypermedia Smart Tutoring Systems improves student learning outcomes; (2) to verify whether the learning outcomes will be grouped into performance clusters (Satisfactory, Good, and Excellent); and (3) to verify whether those clusters will group together the different learning outcomes assessed in four different evaluation procedures. Use of the LMS with hypermedia Smart Tutoring Systems was studied among students of Health Sciences, all of whom had similar test results in the use of metacognitive skills. It explained 38% of the variance in student learning outcomes in the evaluation procedures. Likewise, three clusters that grouped the learning outcomes in relation to the variable ‘Use of an LMS with hypermedia Smart Tutoring Systems vs. No use’ explained 60.4% of the variance. Each cluster grouped the learning outcomes in the different evaluation procedures. In conclusion, LMS with hypermedia Smart Tutoring Systems in Moodle increased the effectiveness of student learning outcomes, above all in the individual quiz-type tests. It also facilitated personalized learning and respect for the individual pace of student-learning. Hence, modules for the analysis of supervised, unsupervised and multivariate learning should be incorporated into the Moodle platform to provide teaching tools that will undoubtedly contribute to improvements in student learning outcomes.

**HIGHLIGHTS**

- Learning management systems (LMS) that incorporate hypermedia Smart Tutoring Systems and personalized student feedback can increase self-regulated learning (SRL).
- Learning management systems with hypermedia Smart Tutoring Systems increased the effectiveness of student learning outcome.
- The use of an LMS with hypermedia Smart Tutoring Systems vs. No use’ explained 60.4% of the variance in student learning outcome.

## Introduction

Over the past decade, a change in the teaching-learning context has been identified. Teacher and student interaction takes places with increasing frequency through learning management systems (LMSs), such as, for example, Moodle (Modular Object Oriented Developmental Learning Environment). Recent studies (Yamada and Hirakawa, 2015; Järvelä et al., 2016) have pointed out that collaborative learning in virtual environments improves learning outcomes. Nevertheless, the mere use of these interactive spaces is not sufficient to ensure that effective learning takes place (Sáiz et al., 2017a). If effective learning is to be guaranteed, the teacher must consider the following points (Mayer, 1998; Clark and Mayer, 2008; de Raadt et al., 2009; Bernard and Bachu, 2015):

(1) The previous concepts of the students in relation to the specific object of learning.
(2) Formulation of the problem in such a way as to help the students structure it in their minds.
(3) The design of strategies for discovery, their breakdown into problem-solving goals.
(4) Data modeling.
(5) The completion of error diagnosis.
(6) The evaluation of the learning process.
(7) Feedback oriented toward processes in learning responses (Hattie and Timperley, 2007).

In summary, LMS, if well-designed, will increase self-regulated learning (SRL), planning, and the use of metacognitive skills. All of those skills will facilitate increased motivation toward learning (Mayer, 1998). Likewise, those learning environments will provide the opportunity for students to develop a framework of key processes, which will foreseeably strengthen effective learning (Sáiz et al., 2017a).

### Feedback and Hypermedia Resources

An LMS facilitates flexible use of hypermedia resources, which helps the teacher to provide both formative and summary feedback, virtually in real time (Hattie and Timperley, 2007). Research in this field (Cerezo et al., 2016) has demonstrated that learning that uses the new Information and Communications Technology (ICT) helps build knowledge. However, for this process to take place, both the declarative and the procedural knowledge of the students must be strengthened through the use of SRL in increasingly challenging tasks (Azevedo, 2005). The stepped structure of the material to learn will assist the preparation of problem-solving strategies in the learner, as the learning goals are sequentially ordered (Winne, 2014; Höök and Eckerdal, 2015); all of which will increase motivation (Zimmerman and Moylan, 2009; Segedy and Biswas, 2015). Nevertheless, so that all of these benefits may be reaped, the use of hypermedia resources has to be included in a dynamic structure that adapts itself to the learning needs of each student to achieve effective learning. In other words, the teacher has to design the architecture of LMS teaching processes. Likewise, an analysis must be done of the different (student–student; student–professor; student–machine) interactions that take place on the platform, so as to redesign, if necessary, processes and procedures. Data-mining techniques are employed in the analysis of those interactions (Educational Data Mining -EDM-) (Romero et al., 2002, 2013). Their automatization was done through modules embedded in the LMS or from the web-service records (logs). Once the *logs* are transferred, the information has to be filtered, selecting only the relevant information that refers to the object of study, as the registers contain a lot of information, not all of which is applicable in each case. The data to be processed is typically represented in JSON (Java Script Object Notation) format (ECMA International, 2017). Once organized, the data may be analyzed by employing EDM techniques from statistical programs such as, for example, SPSS, R, Matlab or through programs that integrate WEKA libraries (in Java) or Pandas (in Python), because the platforms will not usually include complex modules for analysis (Luna et al., 2017).

In summary, the use of hypermedia resources facilitates the development of in-depth and better-quality learning (de Kock, 2016; Norman and Furnes, 2016). Likewise, it increases the use of metacognitive skills (Sáiz and Arnaiz, 2017), as it strengthens planning, supervision, control, and reflection on the object of learning. Active participation by the learner in the learning process is therefore increased. The whole process provides its own feedback in the form of a loop (Zimmerman and Moylan, 2009).

### Intelligent Tutoring Systems and Project Based Learning

Over recent years, work has proceeded along these lines for the design of Artificial Intelligence Systems (Cuba-Ricardo et al., 2015; Li et al., 2015). Those systems include *object level* and *meta-level* processes in the machine following the model of Nelson and Narens (1990). The use of the Project-Based Learning methodology has shown itself to be an effective means of developing those processes in Blended Learning (B-Learning) environments. An intrinsic part of this methodology is the planning and the construction of the learning process through carefully designed research questions (Markham et al., 2003). Recent research (Bannert et al., 2015; Dias et al., 2017) has found that students who use the Project Based Learning methodology in LMS, employ more metacognitive skills (Azevedo et al., 2011; Sáiz and Montero, 2016). A summary of the interaction process is shown in Table 1.

**Table 1:** Cognitive and metacognitive orientation skills in the process of following a Project Based Learning.

Teaching strategies	Student skills
Explanatory strategies	Information analysis (consulting information on the platform)
Control strategies for the acquisition of the explanation (analysis of failure to understand and analysis of the prior knowledge needed to understand the topic)	Reflection on prior knowledge that the material requires and determination of those students who do and those who do not possess that knowledge Analysis of the concepts that have and those that have not been assimilated
Design of practices that support the understanding of theoretical knowledge Feedback from the teacher on the completion of the practice	Completion of practical work Explanation of doubts. Analysis of *feedback*.
Project-Based Learning work, completion of a project based on the application of theoretical knowledge	Completion of the project Explanation of doubts
Continuous *Feedback* throughout the completion of the project (establishment of partial deliveries, revision and *feedback* on them)	Analysis of feedback


Over recent years, many investigations have focused on analyzing the effect of *e-learning* on learning. One relevant aspect is the inclusion of Smart Tutoring System modules. These modules strengthen the personalization of learning and the individualized follow-up of the student, which predicts the learning outcomes by 61.3% (Matwin and Mielniczuk, 2016; Sáiz et al., 2017b).

### Intelligent Tutoring Systems: Virtual Pedagogical Agents

At present, the use of Intelligent Tutoring Systems is increasingly frequent in what are known as Virtual Pedagogical Agents that provide feedback to students in real time on both learning processes and products. These systems facilitate the division of learning into sub-goals and, in consequence, the process of regulating learning. This working methodology can be designed at different levels for pedagogic support to different types of interaction with what is called MetaTutor. A type of tutoring that has the objective of facilitating learning in real time and that is designed to augment the effectiveness of the human tutor (Externally Regulated Learning). There are also different forms of applying this type of intelligent tutoring, which can be done on an individual or a collaborative basis (Collaborative co-Regulated Learning) (Harley et al., 2017).

Along these lines, recent investigations have made it quite clear that Intelligent Tutoring Systems facilitate the development of SRL, because they broaden the skills of reflecting on cognitive and metacognitive processes. Students will not always learn these self-regulated behaviors in a natural way. The use of these systems with students will therefore facilitate:

(1) An understanding of the work.
(2) The establishment of goals and planning
(3) The use of learning strategies.
(4) The adaptation of strategies to goals and planning.

The above-mentioned Intelligent Tutoring Systems include the use of hypermedia systems, that provide feedback on the learning process and support to the student or group of students in real time. In addition, those systems can provide different types of regulation and can predict learning results (Lau et al., 2017).

Likewise, recent students have highlighted that the use of Intelligent Tutoring Systems in learning processes permit personalized instruction and respect the learning rhythms of students (Lajoie and Poitras, 2017). These authors indicate that a lot of data on the learning process is provided through these environments and that their analysis and study through data-mining techniques will provide information for the design of increasingly effective environments and better practices for instruction.

In summary, it may be pointed out that this field of work is in its infancy and, in principle, has broad potential. Nevertheless, variables such as attentional level and self-control, and the use of cognitive and metacognitive skills for learning and motivational and affective factors should be studied in the learners. LMSs, provide a lot of information that is recorded in interaction logs and Educational Data Mining techniques will allow us to analyze such information virtually in real time.

### Application of Data-Mining Techniques for the Analysis of the Results of Interaction on the Learning Management System

**FIGURE 1:**
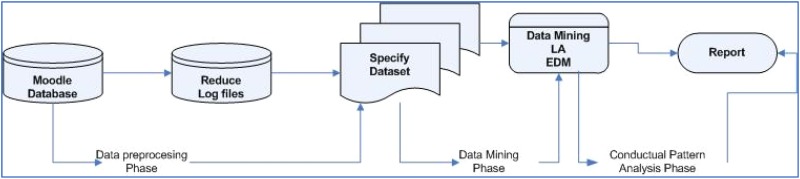
Scheme of the phases in the architecture of the Smart Tutoring System in Moodle.

Another significant aspect in the learning process in LMS is the type of evaluation procedure that is used, because the evaluation procedure appears to be directly related with the learning behavior of the student on the platform (Sáiz and Montero, 2015; Cerezo et al., 2016). As mentioned, EDM techniques are used for the analysis of those behaviors (Romero et al., 2008). EDM techniques can be either supervised learning (classification and regression) or unsupervised learning (*clustering*) (Zacharis, 2015). The results of this analysis may be used to study both the pace and the trajectory of learning of each student. The structure of a personalized Smart Tutoring Systems in Moodle may be consulted in Figure 1. Studies have shown that the use of Smart Tutoring Systems modules are effective at increasing the motivation of students toward learning the subject matter and can thereby achieve effective learning (Kaklauskas et al., 2015).

As previously mentioned, the *Research questions* of this study are:

RQ1: Is the use of LMS with hypermedia Smart Tutoring Systems in Moodle a predictor of student learning outcomes?

RQ2: Will the learning outcomes be structured into different groups by performance when the LMS with hypermedia Smart Tutoring Systems in Moodle is and when it is not being used?

RQ3: Will the cluster groupings differentiate between the results of the different Learning Outcomes?

## Methods

### Participants

A sample of 83 students from the third year of the Degree in Occupational Therapy was used, with 41 subjects in the Control Group (it is the group in which it does not apply Smart Tutoring System in Moodle) and 42 in the Experimental Group (it is the group in which it is applied Smart Tutoring System in Moodle). The descriptive statistics of each group in terms of gender may be seen in Table 2. The assignation of students to either the experimental or to the control group was done by convenience sampling.

**Table 2:** Distribution of the groups and mean and standard deviation for the variables age and gender.

Group		Men			Women		
	*N*	*n*	*M_age_*	*SD_age_*	*n*	*M_age_*	*SD_age_*
Group control	41	7	23.90	2.67	34	22.80	1.66
Group experimental	42	4	24	2.82	38	23.50	6.08


*M_age=_ mean age_,_*SD_*age*_*= standard deviation age.*

### Instruments

#### UBUVirtual Platform, Version 3.1

**FIGURE 2:**
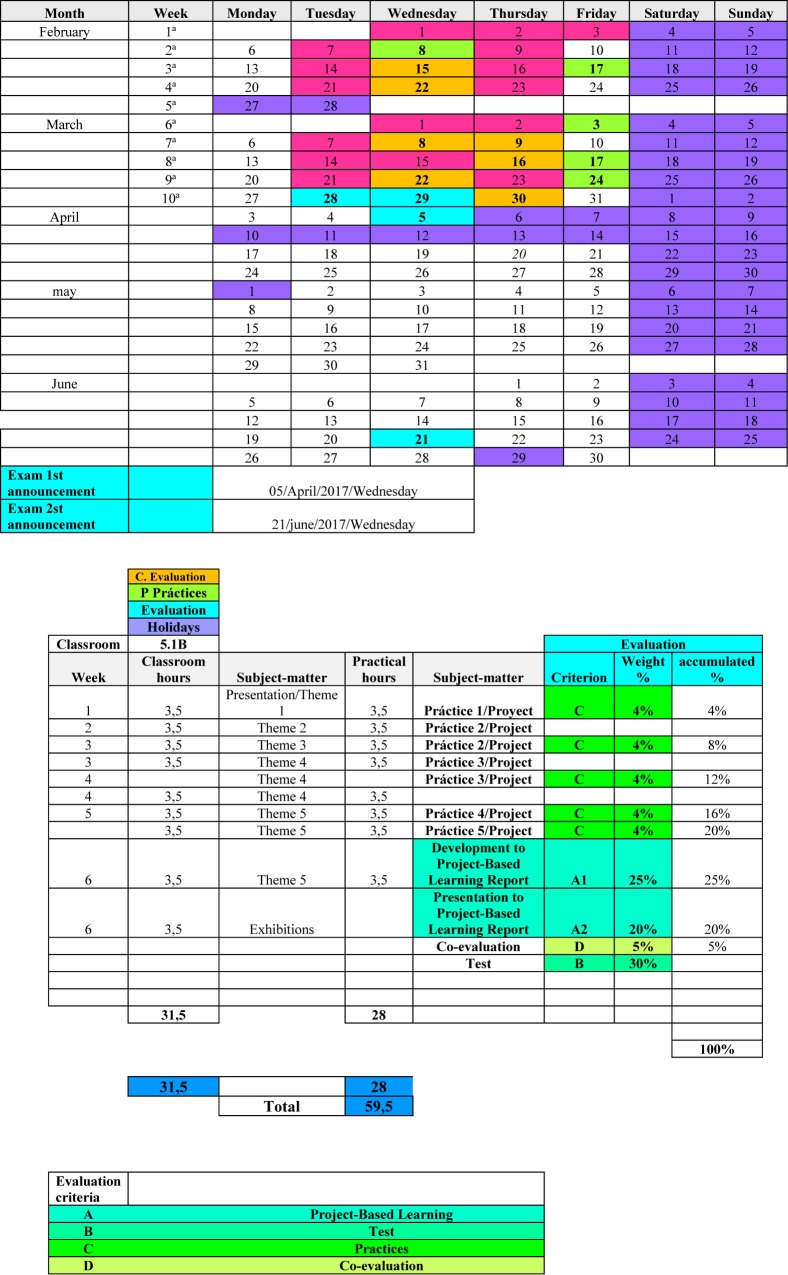
Chronogram of six-monthly activities and process planning.

**FIGURE 3:**
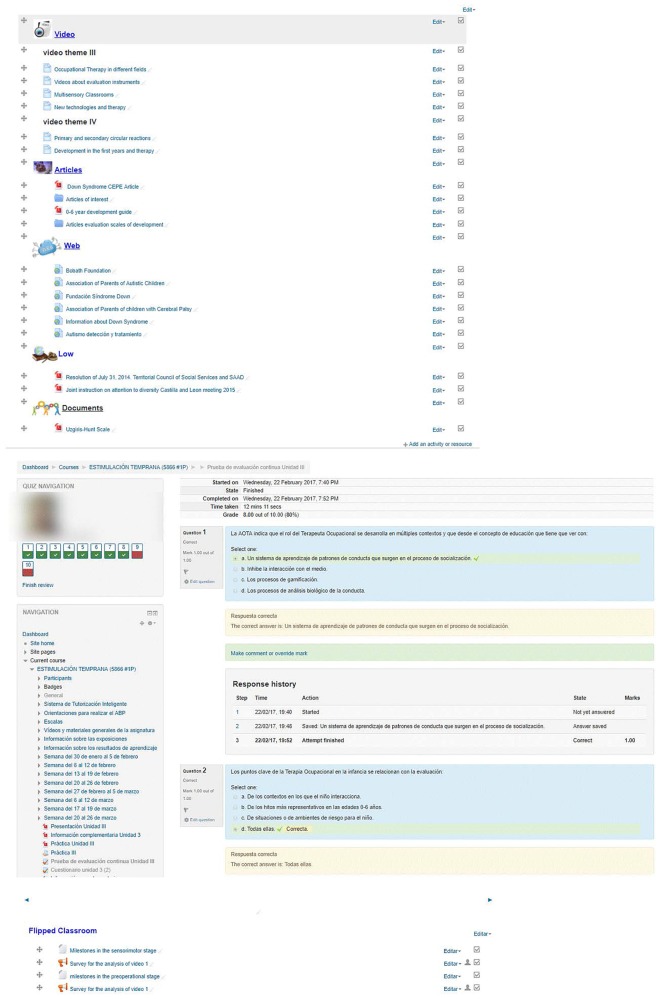
Design of the Moodle platform in the experimental group. Activities were held with network videos, materials, articles and web-based materials of interest. The Flipped Classroom experience included videos prepared *ad hoc* that incorporated quizzes with feedback on the student response.

This platform incorporates a Moodle-based LMS that begins with a constructivist approach, developed through a modular system, that permits progressive configurations. The versatility of modules and their activities facilitate flexible interaction between the users (students and teachers), which is the basis of interactive learning (Saeed et al., 2009). The subject module on which the teaching was developed is based on a Project Based Learning design and systematic structuring of the learning contents, the tasks, and the evaluation procedures. At the beginning of the term, a timetable was made available to the students setting out the learning contents, the weekly activities, the evaluation tests, and the weighting of each test in the final mark (see Figure 2). Likewise, the architecture of the subject may be seen in the LMS. This structure was equally applied in the Control Group and in the Experimental Group, except for the, which were only developed in the Experimental Group. The differences were the use of hypermedia resources: (1) Quizzes with feedback to responses and Flipped Classroom experiences. Comprehension questions were included in these activities, after which feedback was given to the student on the response that had been given (Figure 3).

#### The Learning Skills Scales (ACRAr) of Román and Poggioli (2013)

This instrument has been widely tested in different Spanish-speaking populations (Carbonero et al., 2013). It identifies 32 skills at different points in the processing of information. The skills in each of the scales that constitute the ACRAr are listed in Table 3. Only the Metacognition scale was applied in this scale for which an α = 0.75 was obtained in the sample.

**Table 3:** Skills in each of the ACRAr scales and of the different coefficients of validity.

Scale	Type of skills	Number of skills	Inter-rater reliability	Construct validity	Content validity
Acquisition of information	Repetition and re-reading	6	α = 0.78	*r* = 0.75	*r* = 0.85
Encoding information	Mnemonics, organization, and preparation	12	α = 0.92	*r* = 0.86	*r* = 0.87
Recovery of information	Search and generation of responses	4	α = 0.83	*r* = 0.86	*r* = 0.86
Metacognition	Self-knowledge, self-planning and regulation and self-evaluation	4	α = 0.90	*r* = 0.88	*r* = 0.88
Information processing support	Self-instructions, self-control, counter-distractions, social interventions, intrinsic and extrinsic motivation, and escapist motivations	6	α = 0.90	*r* = 0.88	*r* = 0.88


#### Program of Intervention in the Experimental Group Through an Smart Tutoring Systems in Moodle Architecture

**FIGURE 4:**
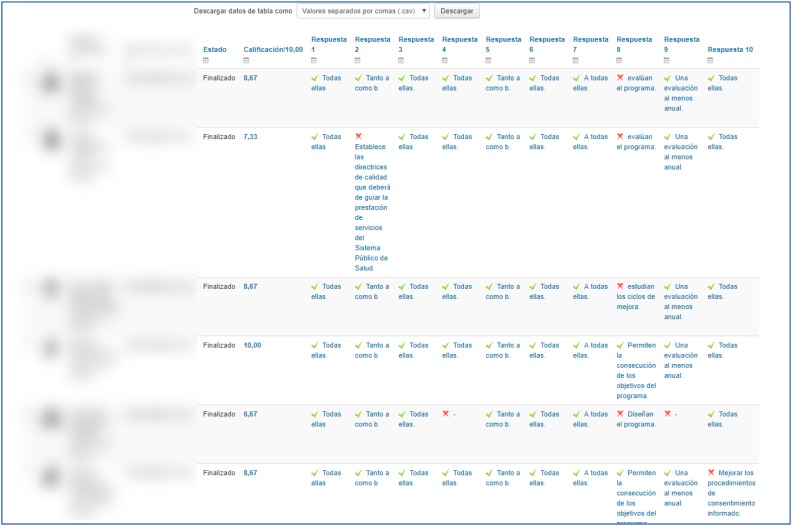
Personalized *feedback* through the quiz on conceptual knowledge. Student responses in the quiz questions receive feedback when errors are detected.

**FIGURE 5:**
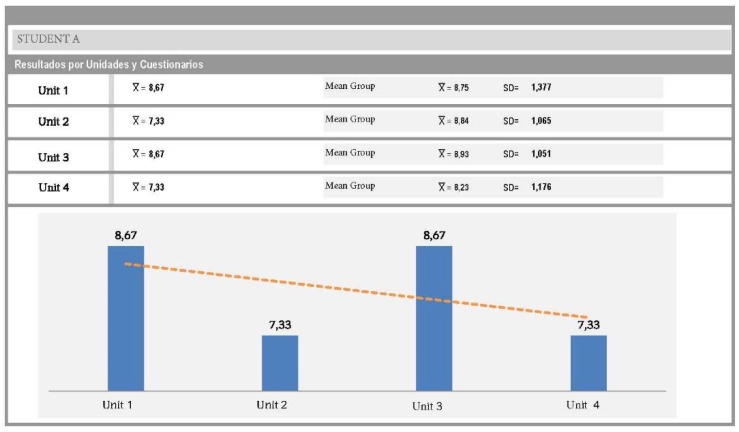
Individualized report following Student A.

**FIGURE 6:**
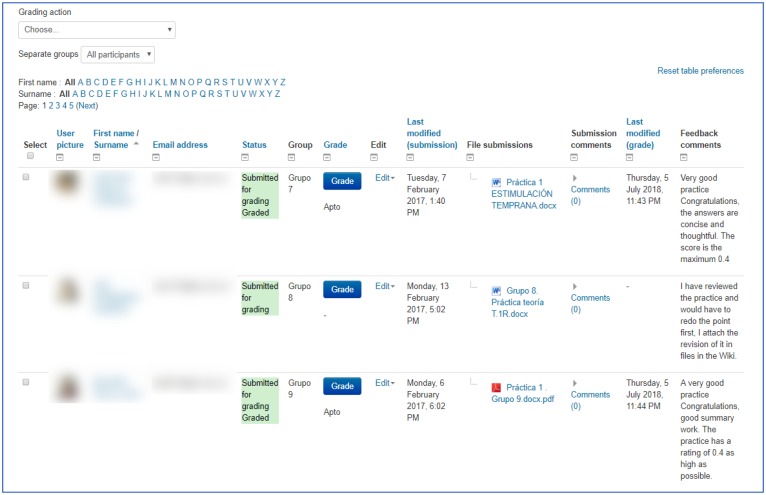
Group feedback on the Moodle platform. Each group of Students uploaded their assignments onto the platform withdate-stamps showing the time and the day of delivery. Likewise, the teacher provided feedback on the process.

An architecture was designed within the Moodle platform with an individual and group tutoring system. The individual tutoring consisted in providing individual feedback for each of the responses that the students gave to each of the five *self-evaluation quizzes* on conceptual knowledge (see Figure 4). In addition, a report was drafted on the performance of each student showing comparisons with the group average of the class (see Figure 5) after the completion of each quiz. Group tutoring was done through the analysis of the group productions in the presentation of the Project Based Learning; an example may be seen in Figure 6.

#### Flipped Learning Experience

Two supporting videos were prepared for the two final units (4 and 5) of the subject module (Sáiz and Arnaiz, 2017). Those videos were used in a Flipped Classroom experience and contained short quizzes to check understanding. Students had to answer the questions to be able to watch the video until the end, and each answer received feedback. These materials are available at the Institutional Repository of the University of Burgos under a Creative Commons license. Only the Experimental Group was affected by this experience.

Unit 4 http://hdl.handle.net/10259/4525 (in Spanish)

Unit 5 http://hdl.handle.net/10259/4526 (in Spanish)

#### Student Learning Outcomes

In Both the Experimental Group and the Control Group Groups were recorded in the following evaluation procedures (see Table 4).

**Table 4:** Distribution of the Evaluation Procedure and percentage of Total Mark.

Name	Percentage of total mark (%)
Learning Outcomes: Self-Evaluation Quizzes	30
Learning Outcomes: Practice	20
Learning Outcomes: Development of Project-Based Learning	25
Learning Outcomes: Presentation of Project-Based Learning	25
Learning Outcomes: Total	100


(1) Learning Outcomes: Self-Evaluation Quizzes with a weight of 30% of the final mark; (2) Learning Outcomes: Practice (practice relating to the theoretical contents of each of the five thematic units) with a weight of 20% of the final mark; (3) Learning Outcomes in Development Project Based Learning with a weight of 25% of the final mark; Learning Outcomes Presentation of Project-Based Learning with a weight of 25% of the final mark; and, finally, Total Learning Outcomes, the final mark of which is the sum of the previously described weightings.

### Procedure

The research project was approved by the Ethics Committee of the University of Burgos. Previously, at the start of the project, the students were informed of the objectives and their participation was at all times on a voluntary basis. Likewise, the informed consent of each participant was recorded in writing. The subject module, for the Control Group and for the Experimental Group, was structured into the following sections on the Moodle Platform: obligatory working material (theory), complementary material, practical activities (five), solution of the Project Based Learning and self-evaluation activities (quizzes) (see Figure 4). Both the practices and the project were done in groups (with either 3 or 5 students).

The difference between GC and GE is found in the use of LMS (see section “Program of Intervention in the Experimental Group Through an Smart Tutoring Systems in Moodle Architecture” and “Flipped Learning Experience”). In both groups, the subject module had a duration of 14 weeks and the type of learning was B-Learning [partly face-to-face and partly through the Moodle Platform]. However, in the Experimental Group, the teaching was structured on the basis of programmed and continuous use of the platform in a Replacement Blend (RB) mode, the interaction fundamentally taking place through deliveries and virtual feedback. A Supplemental Blend (SB) methodology was used in the Control Group, which implied face-to-face feedback. Before starting the teaching program, both groups of students were administered the ACRAr Learning Skills Scale (Román and Poggioli, 2013). The teaching was imparted by the same teacher during the different terms. Convenience sampling was used to assign students to either the Experimental or the Control Group.

### Design, Variables, and Statistical Analysis

These three elements of the study were defined as follows:

1. Designs: a quasi-experimental design was used with a control group equally skilled in the variable metacognitive skills, in order to respond to RQ1. And a descriptive-correlational design was used, to respond to RQ2 and RQ3.
2. Variables: the independent variable was the use of an individual Smart Tutoring Systems in Moodle module (Replacement Blend-RB) and a Flipped Classroom experience v. no experience and the dependent variables were the learning outcomes in different evaluation procedures (see section “Student Learning Outcomes”).

Statistical analyses: (1) analysis of the equivalence between the Control Group and the Experimental Group for the variable metacognitive skills before the intervention, for which the Mann–Whitney *U*-test and the Wilcoxon signed-rank tests were used; (2) analysis of asymmetry and kurtosis; (3) fixed effect ANOVA (use of an Smart Tutoring System in Moodle vs. no use), effect value (eta squared) and the Bonferroni test; (4) Cluster analysis for which the *k-*means clustering technique; (5) Crosstable and (6) Wilk’s Lambda^1^ and Canonical Discriminant Function.

## Results

### Previous Statistical Analysis

Both groups were tested to find out whether they had a similar distribution according to the results of the ACRAr Scale of Metacognitive Skills (Román and Poggioli, 2013) before the study was carried out. To do so, the Mann–Whitney and the Wilcoxon Signed-Rank tests were applied, in which no significant differences were found between both groups in any of the skills (see Table 5), for which reason both groups were considered equivalent. If any differences had been found, this variable would have been considered as a covariable.

**Table 5:** Mann–Whitney *U*-test and Wilcoxon Signed-Rank test between the control group and the experimental group.

Skills	Mann–Whitney *U*-test	Wilcoxon signed rank	*p*
Self-knowledge	435.50	1296.50	0.439
Planning	465.00	765.00	0.711
Self-evaluation	487.00	1348.00	0.945


The indicators of asymmetry and kurtosis were determined, in order to test the characteristics of the distribution of the sample. In asymmetry, the highest values |2.00| indicate extreme asymmetry and the lowest values indicate a normal distribution (Bandalos and Finney, 2001). With regard to the values of kurtosis, values between |8.00|and|20.00| suggest extreme kurtosis (Arias, 2008; Arias et al., 2013). As may be seen in Table 6, the asymmetry and kurtosis values in both groups were within acceptable limits, for which reason a parametric statistic was used.

**Table 6:** Indicators of asymmetry and kurtosis in the Control Group and in the Experimental Group.

	Control group						Experimental group					
Metacognitive skills	*M*	*SD*	*A*	*SEA*	*K*	*SEK*	*M*	*SD*	*A*	*SEA*	*K*	*SEK*
Self-knowledge	19.6	3.74	–1.78	0.37	6.02	0.72	20.33	1.80	–0.94	0.37	3.13	0.72
Planning	12.5	2.71	–0.79	0.37	–0.14	0.72	12.38	2.25	–1.03	0.37	2.01	0.72
Self-evaluation	19.31	2.75	–0.13	0.37	–0.08	0.72	19.4	2.26	–1.12	0.37	2.80	0.72


*M, mean; *SD*, standard deviation; A, asymmetry; *SEA*, standard error of asymmetry; K, kurtosis; SEK, standard error of kurtosis.*

**FIGURE 7:**
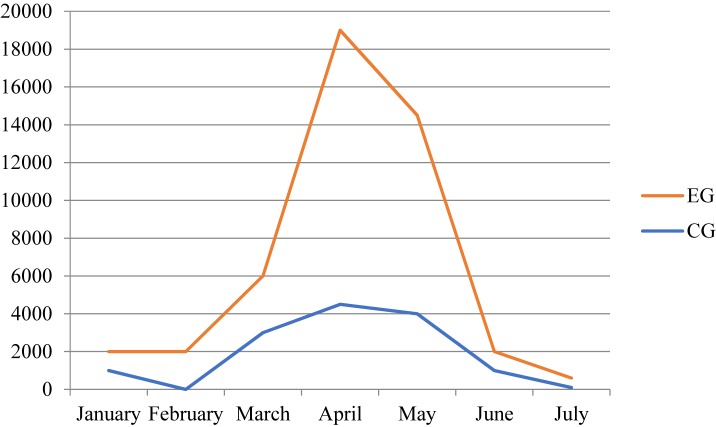
Interaction activity on the UBUVirtual platform in the Experimental Group (EG) and in the Control Group (CG).

The number of records (logs) were also registered: 13.410 in the Control Group and 26.056 in the Experimental Group. These data indicate an increase of 12,646 logs to the platform by the Experimental Group. In other words, the interaction of the Experimental Group students with the platform was almost twice that of the Control Group. The interactions of the teacher numbered 437 with the Control Group and 516 with the Experimental Group, showing an increase in teacher activity of 18%. Student activity in both the Experimental Group and the Control Group is presented below, in Figure 7.

### Confirmation of the Research Questions

A fixed-effects ANOVA (use of LMS with hypermedia Smart Tutoring System vs. no use) was applied to confirm RQ1 (“Is the use of LMS with hypermedia Smart Tutoring Systems in Moodle a predictor of student learning outcomes?”). The results showed that the use of an LMS with hypermedia Smart Tutoring System influenced the learning results of the students in all the evaluation tests, except in the practices, in which both groups of students obtained similar results (see Table 7). The highest effect values in Learning Outcomes: Total and in Learning Outcomes: Self-Evaluation Quizzes explained 38 and 21% of the variance, respectively.

**Table 7:** Single-factor fixed effects ANOVA (use of a Smart Tutoring System in Moodle vs. no use).

	Control group *n* = 41	Experimental group *n* = 42	*F*	*p*	η^2^
	*M (SD)*	*M (SD)*			
(1) Learning outcomes: Practice	2 (–)	2 (–)	–	–	–
(2) Learning Outcomes: Development of Project-Based Learning	2.17 (0.19)	2.24 (0.17)	3.62	0.06	0.04
(3) Learning Outcomes: Presentation of Project-Based Learning	1.70 (0.18)	1.80 (0.14)	8.10	0.006*	0.09
(4) Learning Outcomes: Self-Evaluation Quiz Tests	1.94 (0.32)	2.30 (0.35)	22.62	0.000*	0.21
(5) Learning Outcomes: Total	8.28 (0.62)	9.08 (0.37)	51.32	0.000*	0.38


*Control Group: no use of *Smart Tutoring System in Moodle*; Experimental Group = use of *Smart Tutoring System in Moodle*; *p* = significance; η^*2*^ = eta squared; *M* = mean; *SD* = standard deviation.*

*^∗^*p* < 0.01.*

The unsupervised learning technique was used, in order to test RQ2 (“Will the learning outcomes be structured into different groups by performance when the LMS with hypermedia Smart Tutoring Systems in Moodle is and when it is not being used?”), by grouping the sample of students around different variables, in this case in relation to Learning outcomes. The technique is at present widely used in Educational Data Mining and has shown its effectiveness at ascertaining the characteristics of the groups that yield the best results. It is of assistance to teachers in the improvement of the teaching design (Klösgen and Zytkow, 2002; Muldner et al., 2011; Bogarín et al., 2017, 2018). In particular, the *k-*means clustering was used to test RQ2.

Initially, we use the *k-*means algorithm using as inputs the variables related to learning outcomes. At first using a value of *k* = 2, it was expected that the composition of the two clusters would correspond to that of the two groups: control and experimental. Although practically all the students of the experimental group were grouped in the same cluster (cluster C1), 4 were left out, and in that there were also 20 other students from the control group (see Tables 8, 9).

**Table 8:** Distribution of students in the two clusters in relation to the control and experimental group.

	Cluster		
	C1	C2	Total
No Use of Smart Tutoring System (Control Group)	20	21	41
Use of Smart Tutoring System (Experimental Group)	38	4	42
Total	58	25	83


**Table 9:** Final cluster centers of *k-*means when *k* = 2 is used.

	Cluster	
	C1 *n* = 58	C2 *n* = 25
Learning Outcomes: Development of Project-Based Learning	2.02	2.29
Learning Outcomes: Presentation of Project-Based Learning	1.74	1.94
Learning Outcomes: Self-Evaluation Quizzes	1.81	2.27
Learning Outcomes: Total	7.81	9.03


In a second step, a value of *k* = 3 was used, and this time, the clusters were more compact and interpretable (see Tables 10, 11). Cluster C3, which we could associate with the group of excellent students, contains the bulk of the students of the experimental group and some students of the control group (sometimes the personal aptitudes of a student make their learning results good, regardless of the teaching technique used). Cluster C2, which we could associate with good students, contains the rest of the students of the experimental group and the bulk of students in the control group. Finally, in cluster C1, which could be matched with less bright students, there are only seven students and they are all from the control group.

**Table 10:** Distribution of students in the three clusters in relation to the control and experimental group.

	Cluster case number			Total
	C1	C2	C3	
No Use of Smart Tutoring System	7	22	12	41
Use of Smart Tutoring System	0	7	35	42
Total	7	29	47	83


**Table 11:** Final cluster centers of *k*-means when *k* = 3 is used.

	Cluster		
	C1 (sufficient) *n* = 7	C2 (good) *n* = 29	C3 (excellent) *n* = 47
Learning Outcomes: Development of Project-Based Learning	2.00	2.11	2.30
Learning Outcomes: Presentation of Project-Based Learning	1.53	1.84	1.95
Learning Outcomes: Self-Evaluation Quizzes	1.53	1.95	2.33
Learning Outcomes: Total	7.04	8.29	9.13


**FIGURE 8:**
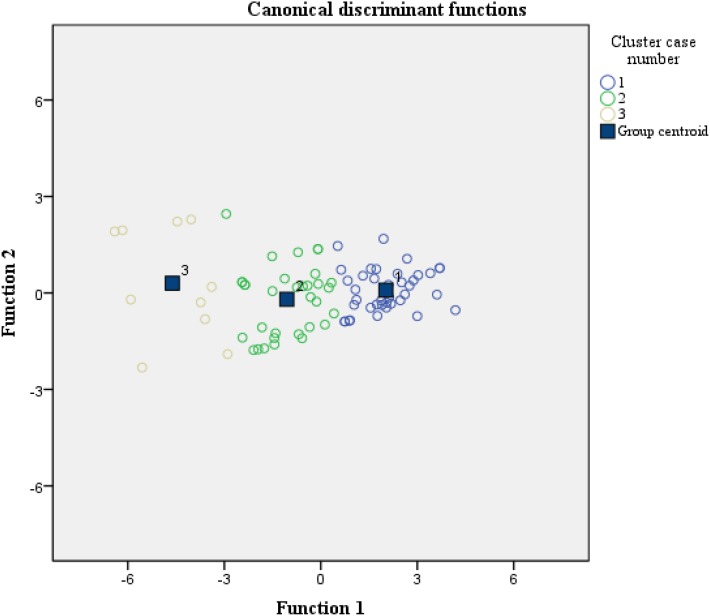
Canonical discriminant function in the three clusters.

Also, a discriminant analysis was conducted to study RQ3: “Will the cluster groupings differentiate between the results of the different Learning Outcomes?” The results pointed to a different behavior of the three clusters in the different evaluation procedures. Nevertheless, Wilks’ Lambda was only of statistical significance in Learning Outcomes: Total (see Table 12). The behavior of the three clusters is shown below in Figure 8.

**Table 12:** Discriminant analysis between groups.

	*Wilks’ Lambda*	*ASE Lambda*	*T*	*p Lambda*
Learning Outcomes: Development of Project-Based Learning	0.082	0.072	1.09	0.272
Learning Outcomes: Presentation of Project-Based Learning	0.131	0.078	1.593	0.111
Learning Outcomes: Self-Evaluation Quizzes	0.039	0.034	1.143	0.253
Learning Outcomes: Total	0.063	0.027	2.307	0.021^∗^


*ASE, approximate standard error.*

*^∗^*p* < 0.05.*

## Discussion and Conclusion

The fact that the clusters obtained by *k-*means, when using as input variables the learning outcomes, have this strong correspondence with the control and experimental groups is an additional indication that the use of Smart Tutoring System seems to increase learning outcomes in the students. A possible explanation is that the system helps to apply the metacognitive skills of orientation, planning, evaluation, and reflection to problem-solving tasks, which helps to define the problem-solving process through graduated steps of progressive difficulty (Azevedo, 2005; Winne, 2014; Höök and Eckerdal, 2015; Cerezo et al., 2016; Harley et al., 2017). Moreover, this process facilitates SRL (Cerezo et al., 2016; Sáiz and Montero, 2016; Lau et al., 2017) and the personalized feedback of the teacher in real time, which increases the motivation of the student toward the learning material (Hattie and Timperley, 2007; Zimmerman and Moylan, 2009; Segedy and Biswas, 2015). The use of LMS that incorporate hypermedia Smart Tutoring Systems includes all these characteristics in the platform for the strengthening of *object-level* and *meta-level* structures (Cuba-Ricardo et al., 2015; Li et al., 2015). The effectiveness of this system architecture is complemented through the use of the Project-Based Learning methodology on the Moodle Platform (Bannert et al., 2015). It all means that the problem may be solved through progressive approximations to the goal (Azevedo et al., 2011) and it favors the use of metacognitive skills of planning and evaluation applied to both process and product in the learning activity (Sáiz and Montero, 2015). Likewise, if this form of personalized education in B-Learning environments is supported by the use of hypermedia resources, such as for example Flipped Classroom experiences (Sáiz and Arnaiz, 2017; Wang, 2017) and *quizzes* with interactive *feedback* on the responses in real time (Sáiz et al., 2017b), its effectiveness is all the greater. Therefore, the personalization of learning together with the use of the previously described methodological and technological resources is in step with the learning rhythm of the student (Matwin and Mielniczuk, 2016).

In summary, if the B-Learning environments use the LMS that incorporate hypermedia Smart Tutoring Systems, they appear to be more effective (Sáiz et al., 2017b). In addition, the student learning outcomes in different evaluation procedures appear to be related with the use of those modules, the ones that explain 57.8% of the variance in the learning outcomes, especially those related with the completion of self-evaluation *quizzes*. One explanation may be that those systems allow for individualized student follow up and that the individualized feedback strengthens the development of *insight* throughout the learning process. Another important preventive measure to identify at-risk students is to find the groupings in clusters, as they explain 60.4% of the variance in the learning outcomes. A map can be sketched from an analysis of those clusters for the prediction of performance in the various evaluation procedures. All of the above will foreseeably allow the correction of possible learning problems and thereby reinforce higher indicators of academic performance (Díez et al., 2017).

One possible explanation is that the LMS with hypermedia Smart Tutoring Systems permit the development of greater personalized learning that is more in keeping with the pace of learning of each student. In addition, the records that the interactions between those learners leave on the system permit a lot of information to be gathered that can be analyzed by using data-mining techniques. The teacher is therefore able to access information in real time that helps with the systematic regulation throughout the teaching-learning process practically in real time. Hence, strengthening the incorporation of analytical tools in Moodle that can generate automatic (supervised and unsupervised) learning techniques and multivariate analysis techniques in an easy way is an important issue for those in charge of universities. It will provide an analysis for the teacher in real time of the learning characteristics of their students throughout both the teaching and the learning process. It will also permit the teacher to ascertain the grouping around variables of both performance and learning behaviors that are recorded on the platform from the start of the course (Bogarín et al., 2017, 2018). All this information will facilitate the adjustment of the teaching to the needs of each student and of each one of the groups found with these clustering techniques that can detect similar groups in relation to a series of variables that the teacher may *a priori* define as significant, all of which will foreseeably increase the results of effective learning (Sáiz, 2017, unpublished).

### Limitations of This Study and Future Lines of Investigation

The conclusions of this study must be analyzed with caution with regard to any generalization of the results, due to various reasons such as the size of the sample and the origin of the students (from the same university and the same degree course), sample characteristics and type of sample. Future investigations will therefore be directed at enlarging the size of the sample and the number of degree courses.

## Ethics Statement

The Ethics Committee of the University of Burgos approved this study. Written informed consent was obtained from all participants.

## Author Contributions

MCS-M performed the statistical analyses and data interpretation and prepared the manuscript. JFD-P and CIG-O supervised the statistical analyses and collaborated in the drafting of the conclusions of the paper. RM-S supervised the document structure, analyses, and results.

## Conflict of Interest Statement

The authors declare that the research was conducted in the absence of any commercial or financial relationships that could be construed as a potential conflict of interest.

## Footnotes

1 Wilk’s Lambda tests how well each level of independent variable contributes to the model. The scale ranges from 0 to 1, where 0 means total discrimination, and 1 means no discrimination.
